# Probe-integrated electrochemical immunosensor based on electrostatic nanocage array for reagentless and sensitive detection of tumor biomarker

**DOI:** 10.3389/fchem.2023.1121450

**Published:** 2023-03-10

**Authors:** Dong Chen, Xuan Luo, Fengna Xi

**Affiliations:** ^1^ General Surgery Department, Shanxi Bethune Hospital, Taiyuan, China; ^2^ Department of Chemistry, Zhejiang Sci-Tech University, Hangzhou, China

**Keywords:** tumor biomarker, electrochemical immunosensor, electrostatic nanocage array, confined redox probe, reagentless detection

## Abstract

Sensitive detection of tumor biomarkers is crucial for early diagnosis and prognosis evaluation of cancer. Owing to no need of labelled antibody, formation of sandwich immunocomplexes and additional solution-based probe, probe-integrated electrochemical immunosensor for reagentless detection of tumor biomarkers is highly desirable. In this work, sensitive and reagentless detection of a tumor biomarker is realized based on fabrication of a probe-integrated immunosensor by confining redox probe in electrostatic nanocage array modified electrode. Indium tin oxide (ITO) electrode is employed as the supporting electrode because it is cheap and easily available. The silica nanochannel array consisted of two layers with opposite charges or different pore diameters was designated as bipolar films (bp-SNA). In this work, Electrostatic nanocage array is equipped on ITO electrode by growth of bp-SNA with two layered nanochannel array having different charge properties including a negatively charged silica nanochannel array (n-SNA) and a positively charged amino-modified SNA (p-SNA). Each SNA can be easily grown with 15 s using electrochemical assisted self-assembly method (EASA). Methylene blue (MB) is applied as the model electrochemical probe with positive charge to be confined in electrostatic nanocage array with stirring. The combination of the electrostatic attraction from n-SNA and the electrostatic repulsion from p-SNA endows MB with highly stable electrochemical signal during continuous scanning. When the amino groups of p-SNA are modified using the bifunctional glutaraldehyde (GA) to introduce aldehydes, the recognitive antibody (Ab) of the most commonly used tumor biomarker, carcinoembryonic antigen (CEA), can be covalently immobilized. After the non-specific sites are blocked, the immunosensor is successfully fabricated. As the formation of antigen-antibody complex decreases electrochemical signal, the immunosensor can achieve reagentless detection of CEA ranged from 10 pg/mL to 100 ng/mL with a low limit of detection (LOD, 4 pg/mL). Determination of CEA in human serum samples is realized with high accuracy.

## 1 Introduction

Cancer, malignant tumor, is a serious threat to human health. As known, early screening and diagnosis of cancer is the key to reduce mortality. Tumor biomarkers are substances secreted by tumor cells or produced by the body to resist tumors in the process of tumor formation and growth (Fumet et al., 2020; Jones et al., 2021; Liu et al., 2022) Their appearance can precede the morphological and biological changes of cells or tissues. Thus, tumor biomarkers play an important role in the pathological diagnosis, accurate classification, clinical diagnosis and treatment, observation of curative effect, and prognosis evaluation of cancer. For example, carcinoembryonic antigen (CEA) is one of the most important tumor biomarkers related to colon cancer, lung cancer and ovarian cancer (Tan et al., 2009; Grunnet and Sorensen, 2012; Sorensen et al., 2016) CEA is usually produced in gastrointestinal tissues during fetal development, but it stops after birth. Commonly, CEA exists in the blood of healthy adults at a very low level (usually below 5.0 ng/mL). However, it increases significantly in cancer patients. Therefore, rapid and sensitive determination of CEA is of great significance.

At present, methods based on enzyme linked immunosorbent assay (ELISA) (Arnab et al., 2014) chemiluminescence (Hou et al., 2022) fluorescence (Wu et al., 2021) surface plasmon resonance (SPR) (Liu et al., 2016) and quartz crystal microbalance (QCM) (Chi et al., 2020) have been used for CEA detection. However, these methods commonly suffer from low operation speed, unsatisfied sensitivity, expensive instruments, and special reagents. Electrochemical (EC) immunosensors are attractive because of simple instrument, easy operation, high sensitivity and great potential for integration and miniaturization (Lin et al., 2020; Zhang et al., 2022a) In addition, electrochemical sensors can achieve high sensitivity or even ultra-high sensitivity in combination with nanomaterial-based signal amplification (Ayl´en et al., 2022; Zheng et al., 2022a) These merits of characteristics exhibit great potential in fast and sensitive detection of tumor biomarkers in biological samples. Usually, EC detection is based on the electrochemical signal of the analyte or redox probe (Gong et al., 2022a; Zheng et al., 2022b) As tumor biomarkers are proteins with no electrochemical activity, electrochemical detection of tumor markers is mainly realized based on the change of the electrochemical signal of redox probes (Chen et al., 2022) Therefore, effective signal probes are the key to detect tumor biomarkers in EC immunoassay. Compared with the detection based on free probes in solution, the probe-integrated (reagentless) immunosensors based on the immobilization of redox probes on the electrode surface can realize convenient and rapid detection (Gong et al., 2022b) In comparison with the sandwich immunoassay, these label-free immunosensors without label of antibody also greatly reduce the cost of detection and simplify the operation steps. Thus, the development of simple and reliable probe-integrated electrochemical immunosensors is highly desirable.

The introduction of nanomaterials to efficiently immobilize signal probes is the key to build probe-integrated immunosensors with high sensitivity. Recent studies have proven that silica nanochannel array film (SNA), also vertically-ordered silica nanochannels film (VMSF), is attractive as a matrix to enrich electrochemical or electrochemiluminescence probes (Liang et al., 2021; Ma et al., 2022a) SNA is a nanoscale ultrathin film (20–200 nm) with high pore density (∼7.5 × 10^12^ pores/cm^2^), ultrasmall (2–3 nm) nanochannel array (Walcarius, 2021; Yan et al., 2021) These unique structures endow SNA modified electrodes with significant advantages in electrochemical sensing applications (Ma et al., 2022b; Huang et al., 2022; Zou et al., 2022) For instance, ultrasmall nanochannels have screening capabilities on molecular level. On the one hand, the large substances in complex matrices cannot enter the nanochannels, leading to high anti-fouling of the electrodes (Zhou et al., 2022a; Zhou et al., 2022b; Deng et al., 2023) On the other hand, the dissociation of silanol groups (Si-OH, p*K*_a_∼2) in SNA makes the surface of nanochannel negatively charged, which can enrich positively charged molecules through electrostatic interaction (Zheng et al., 2022c; Wang et al., 2022; Huang et al., 2023) The charge of SNA can also be reversed by introducing functional groups. Thus, functional nanostructures, e.g., electrostatic nanocage arrays, can be flexibly designed by growing multilayer of SNA (Gong et al., 2022b; Gong et al., 2022c) Secondly, SNA with confined probe can generate gating signal when biomacromolecules are detected. As known, the SiO_2_ structure of SNA is an electrical insulator. As antibodies cannot enter the nanochannels of SNA, they can only be immobilized on the outer surface of SNA. When tumor biomarkers bind with the recognitive antibodies, the entrance of some nanochannels will be blocked, decreasing the signal of immobilized electrochemical probes resulting from the increased interface resistance of electrode (Gong et al., 2022b) Therefore, in combination of high specific surface area, SNA-based electrodes exhibit great potential for the construction of probe-integrated electrochemical immunosensors.

In this work, a probe-integrated electrochemical immunosensor is fabricated based on confinement of electrochemical probe in electrostatic nanocage array prepared by double-layered bipolar SNA (bp-SNA), which enables sensitive and reagentless detection of tumor marker carcinoembryonic antigen (CEA). The cheap and easily available indium tin oxide (ITO) electrode is applied as the supporting electrode to successively grow the negatively charged SAN (n-SNA) and the positively charged SNA with rich amine groups (p-SNA). Owing to the asymmetric surface charge, n-SNA and p-SNA constitute bp-SNA with electrostatic nanocage array. The electrochemical probe, methylene blue (MB), is electrostatically confined in the nanocage array to achieve high loading and good stability (MB@bp-SNA/ITO). After the amine groups of p-SNA is modified to produce aldehyde surface, CEA recognitive antibody (Ab) are covalently immobilized on the outer surface of bp-SNA followed by blocking non-specific sites to fabricate the immunosensor interface. The constructed probe-integrated immunoassay system is also employed to achieve rapid and highly sensitive detection of CEA. The developed probe-integrated electrochemical immunosensor has advantages of simple fabrication and easy operation because of no need labelled antibody, formation of sandwich immunocomplexes and additional solution-based probe. In addition, stable enrichment of electrochemical probe in electrostatic nanocage array leads to reagentless detection of tumor biomarkers with high sensitivity and good stability.

## 2 Materials and methods

### 2.1 Chemicals and materials

Tetraethyl orthosilicate (TEOS), hexadecyl trimethyl ammonium bromide (CTAB), sodium phosphate dibasic dodecahydrate (Na_2_HPO_4_•12H_2_O), methylene blue trihydrate (MB), glutaraldehyde (GA) and tripyridine ruthenium chloride hexahydrate (Ru(bpy)_3_Cl_2_•6H_2_O) were purchased from Aladdin Chemistry Co., Ltd. (Shanghai, China). Sodium dihydrogen phosphate dehydrate (NaH_2_PO_4_•2H_2_O), 3-aminopropyltriethoxysilane (APTES) and potassium hydrogen phthalate (KHP) were purchased from Macklin (Shanghai, China). Ethanol (EtOH) and hydrochloric acid (HCl) was obtained from Hangzhou Gaojing Chemistry Co., Ltd. (Hangzhou, China). Sodium nitrate (NaNO_3_) were purchased from the Wuxi Zhang Wang Chemical Industry (Wuxi, China). Carbohydrate antigen 199 (CA199), carcinoembryonic antigen (CEA), CEA antibody, prostate specific antigen (PSA), carcinoma antigen 125 (CA125) and alphafetoprotein (AFP) were purchased from Beijing KEY-BIO Biotech Co., Ltd. (Beijing, China). ITO coated glasses (<17 Ω/square, thickness: 100 ± 20 nm) were purchased from Zhuhai Kaivo Optoelectronic Technology (Shenzhen, China). To get a clean surface, ITO was immersed in NaOH solution (1 M) overnight and then sonicated in acetone, ethanol, and ultrapure water, respectively. All aqueous solutions were prepared with ultrapure water (18.2 MΩ cm) in this work.

### 2.2 Measurements and instrumentations

The morphology of bp-SNA modified electrode were investigated using field-emission scanning electron microscope (SEM, S-4800, Hitachi, Japan) and transmission electron microscope (TEM, Hitachi, HT7700, Japan), respectively. The acceleration voltage of TEM is 100 kV. The TEM sample was prepared by peeling the bp-SNA from the ITO electrode surface. After being dispersed in ethanol (200 μL) and sonicated, the obtained solution was dropped onto a copper grid. An Autolab PGSTAT302N electrochemical workstation (Metrohm, Switzerland) was used to perform all electrochemical measurements including cyclic voltammetry (CV), electrochemical impedance spectroscopy (EIS) and differential pulse voltammetry (DPV). A traditional three-electrode system was used with bare or modified ITO as working electrode, Ag/AgCl (saturated KCl solution) as reference electrode and Pt wire electrode as counter electrode. The applied DPV parameters including step modulation amplitude (0.005 V), modulation time (0.05 s) and interval time (0.2 s).

### 2.3 Preparation of MB@bp-SNA/ITO

The n-SNA was firstly grown on bare ITO electrode (1 cm × 0.5 cm) by EASA method (Zhang et al., 2022b; Yang et al., 2022) Briefly, a mixture consisting of NaNO_3_ aqueous solution (20 mL, 0.1 M, pH = 2.6), ethanol (20 mL), TEOS (3.050 mL), and CTAB (1.585 g) were prepared. After stirring for 2.5 h to pre-hydrolyze TEOS, the precursor for the growth of n-SNA was obtained. Then, bare ITO electrode was immersed into the precursor solution and applied a constant current density (−0.70 mA cm^−2^) for 10 s. After being quickly rinsed with ultrapure water and aged at 120°C overnight, the modified electrode containing surfactant micelles (SM) inside the nanochannels was obtained and termed as SM@n-SNA/ITO. The bp-SNA/ITO electrode was then obtained after p-SNA was grown on n-SNA/ITO electrode by EASA method. Typically, a solution consisting of NaNO_3_ aqueous solution (20 mL, 0.1 M, pH = 2.6), ethanol (20 mL), TEOS (2.732 mL), APTES (0.318 mL) and CTAB (1.585 g) were mixed and stirred for 2.5 h to obtain the precursor solution. Before the growth of p-SNA, the possible silica spheres on SM@n-SNA/ITO electrode were removed by scotch tape. SM could be removed by immersing the SM@n-SNA/ITO electrode in HCl-ethanol solution (0.1 M) and stirring for 5 min. Subsequently, the n-SNA/ITO was placed in the above precursor solution and a constant current density (−0.70 mA cm^−2^) was applied to the electrode for 15 s. After rinsed with a large amount of ultrapure water and aged overnight at 120°C, the SM@bp-SNA/ITO electrode was obtained. Finally, silica spheres on the surface of bp-SNA/ITO electrode were removed by Scotch tape. SM was removed using HCl-ethanol solution (0.1 M) to get an electrode with electrostatic nanocage array, that was termed as bp-SNA/ITO. Then, MB was confined in electrostatic nanocage array. Briefly, bp-SNA/ITO was immersed in a PBS (0.01 M, pH = 7.4) solution containing MB (1 mM) under stirring for 6 min. After thorough rise, electrode with confined probe was obtained and denoted as MB@bp-SNA/ITO.

### 2.4 Fabrication of immunosensor

To fabricate the immuno-recognitive interface, glutaraldehyde (GA) was chosen as the bifunctional linker to introduce aldehyde groups on the outer surface of p-SNA. Briefly, the MB@bp-SNA/ITO electrode was immersed in GA (1%, pH = 7.4) solution for 1 h at 37°C in the dark. After removing the residual GA, CEA antibody (50 μL, 10 μg/mL) was dropped on the surface of GA/MB@bp-SNA/ITO electrode and incubated at 37°C for 1 h. The unbounded CEA antibody was rinsed with PBS and the obtained electrode (Ab/GA/MB@bp-SNA/ITO) was immersed in BSA (1 mg/mL in 0.01 M PBS, pH 7.4) at 37°C for 1 h to block non-specific sites. Finally, the immunosensor, BSA/Ab/GA/MB@bp-SNA/ITO electrode, was obtained.

### 2.5 Electrochemical detection of CEA

For the detection of CEA, each BSA/Ab/GA/MB@bp-SNA/ITO electrode was incubated with different concentrations of CEA at 37°C for 0.5 h. PBS (0.01 M, pH = 7.4) was used as the detection electrolyte and the electrochemical signals of MB in absence and presence of CEA were measured by DPV. For the analysis of real sample, human serum (healthy woman) was diluted by a factor of 50 using PBS (0.01 M, pH 7.4).

## 3 Results and discussion

### 3.1 Fabrication of probe-integrated immunosensor based on confinement of redox probe on electrostatic nanocage array

**FIGURE 1:**
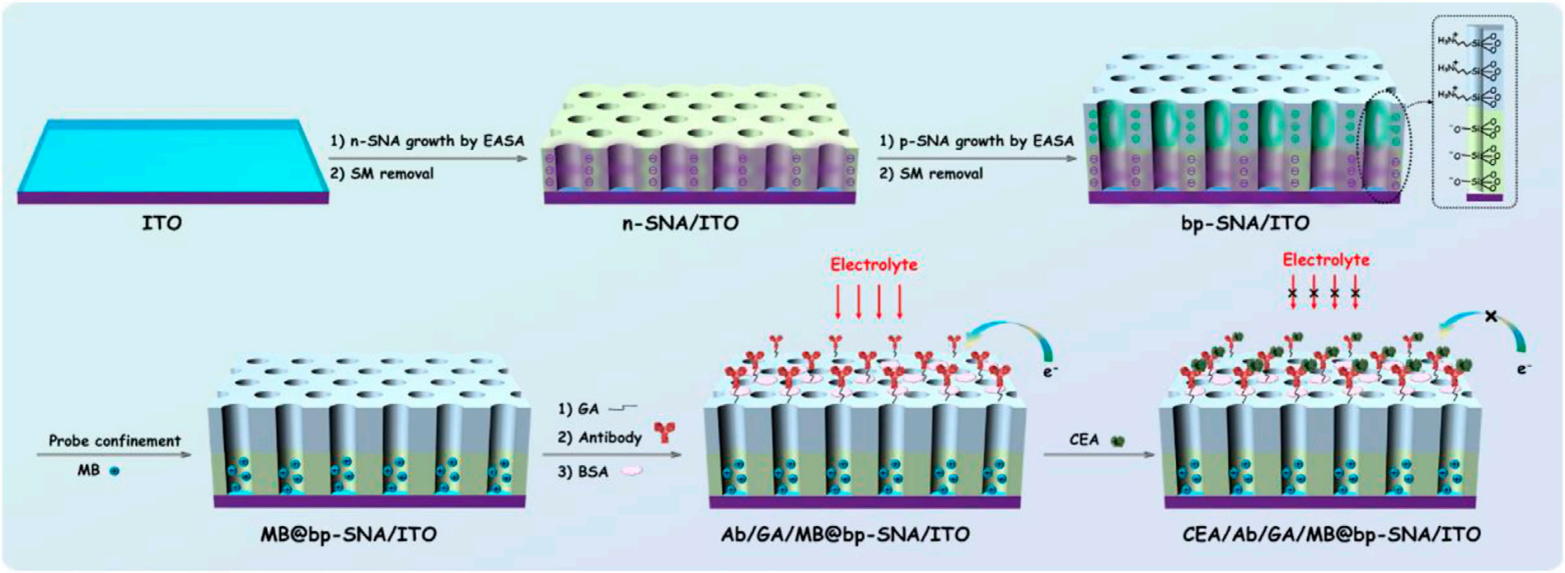
Schematic illustration for the fabrication of probe-integrated immunosensor based on MB confinement in bp-SNA and the electrochemical detection of CEA.

Electrochemical detection has the advantages of fast detection speed, easy miniaturization and integration. In addition, high sensitivity can be achieved by combining nanomaterial for signal amplification. As the electrode is easy to be contaminated by the complex matrix of real sample, the current electrochemical immunoassay mostly uses sandwich immune complexes with the labeling signal on the second antibody (Ab_2_) for detection. However, antibody labeling and sandwich immunoassay mode increase the operation complexity and detection cost. Electrochemical immunoassay without the label of antibody remains challenges. In this work, a probe-integrated electrochemical immunosensor is constructed for highly sensitive detection of CEA by confinement redox probe on electrostatic nanocage array. Figure 1 illustrates the fabrication of probe-integerated immunosensor based on confinement of redox probe on electrostatic nanocage array. As shown, the cheap ITO electrode is used as the support electrode and SNA with negative charge (n-SNA) can be rapidly grown on ITO surface within 10 s by electrochemical assisted self-assembly method (EASA). Using the negative surface charge and high specific surface area of n-SNA, small molecule probes with opposite charges (MB) can be immobilized in large quantities through electrostatic interaction. However, the probes are easy to run off during use, thus reducing the detection accuracy of immunoassay. In this work, positive SNA (p-SNA) is further grown on the surface of n-SNA, and a bipolar film (bp-SNA) is prepared by using the above double-layered SNA with asymmetric surface charge. As shown, bp-SNA is consisted of electrostatic nanocage array. Based on the electrostatic attraction of the inner n-SNA layer and the electrostatic repulsion of the outer p-SNA layer, the electrostatic nanocage array can achieve high loading and high stability confinement of the positive electrochemical probe MB (MB@bp-SNA/ITO).

Using functional siloxane, SNA with reactive groups (e.g., amino group, epoxy group, etc.) can be introduced. Herein, amino containing siloxanes (3-aminopropyl-triethoxysilane, APTES) is employed to grow p-SNA with rich amino groups on n-SNA. After the amino group on the outer surface of p-SNA reacts with glutaraldehyde (GA/MB@bp-SNA/ITO), the introduced aldehyde group can be applied to covalently immobilize the recognitive antibody of CEA. After the non-specific sites are blocked by bovine serum albumin (BSA), the immunosensor can be fabricated and donated as BSA/Ab/GA/MB@bp-SNA/ITO. When CEA specifically binds to the Ab on the immune recognitive interface, the formed antigen-antibody complex increases the interface resistance of the electrode, resulting in the reduction of the electrochemical signal of MB. Based on this mechanism, the electrochemical detection of CEA can be realized.

### 3.2 Morphology of bp-SNA

**FIGURE 2:**
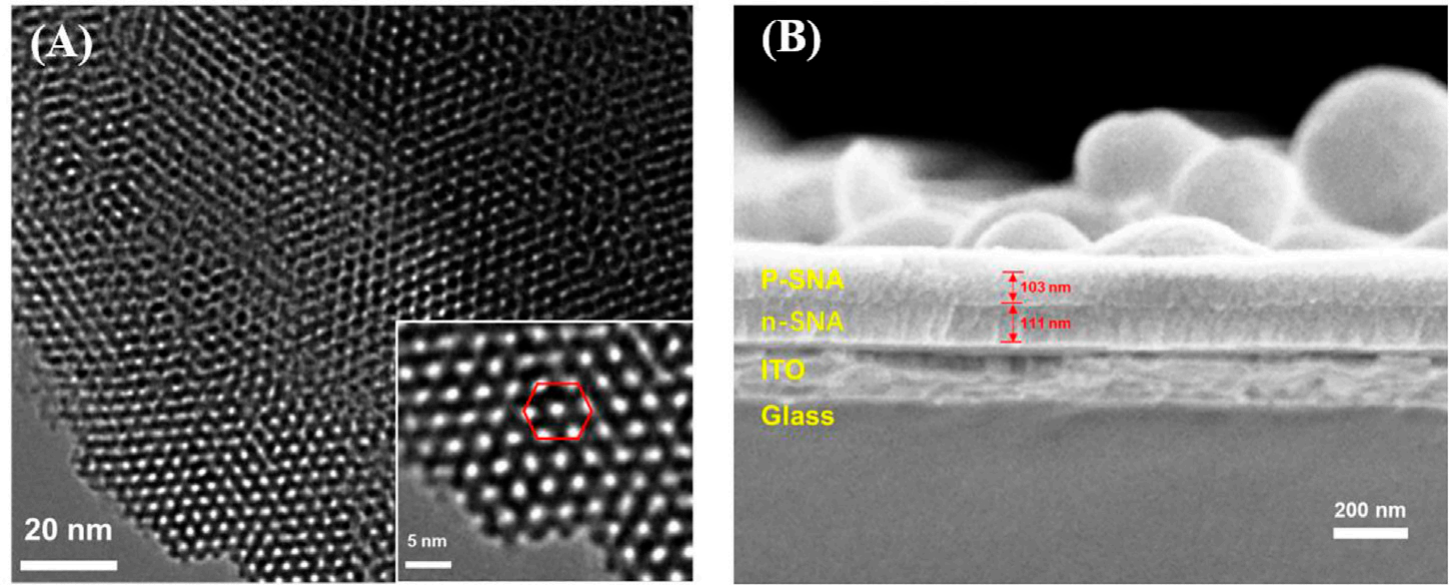
**(A)** Top-view TEM images of bp-SNA at different magnification. **(B)** Cross-sectional SEM image of bp-SNA/ITO.

Transmission electron microscope (TEM) and scanning electron microscope (SEM) are applied to characterize the morphology of bp-SNA. From the top-view TEM images at different magnification, it can be seen that the bp-SNA has nanochannel array evenly arranged in hexagonal shape. No obvious defects are observed and the diameter of each channel is 2.3 ± 0.3 nm (*n* = 11) (Figure 2A). The cross-sectional SEM image reveals p-SNA layer, n-SNA layer, ITO layer and glass from top to bottom (Figure 2B). The thicknesses of p-SNA layer and n-SNA are 103 ± 1 nm (*n* = 3) and 111 ± 1 nm (*n* = 3), respectively. The silica spheres adhered on the surface can be easily removed by adhesive tap.

### 3.3 Stable confinement of MB in bp-SNA

**FIGURE 3:**
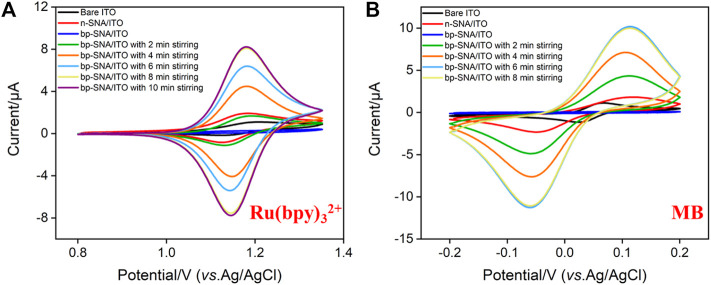
CV curves obtained on bare ITO, n-SNA/ITO, bp-SNA/ITO electrodes in KHP (0.05 M, pH = 4) containing 10 μM Ru(bpy)_3_^2+^ **(A)** or MB **(B)** solution.

The standard electrochemical probe (Ru(bpy)_3_^2+^) was used to investigate the changes of electrode surface during the electrode modification. As shown in Figure 3A, the redox signal of Ru(bpy)_3_^2+^ can be observed on bare ITO electrode. When n-SNA was grown on ITO surface, a significantly increased redox peak was observed. This is due to the negative charge generated by the ionization of silanol group (p*K*_a_∼2) in n-SNA, which can attract Ru(bpy)_3_^2+^ electrostatically, thus generating high redox signal. However, there is no obvious redox peak on bp-SNA/ITO after further growth of p-SNA, which is attributed to the electrostatic repulse from the positively charged sites generated after the protonation of amino groups in p-SNA. When bp-SNA/ITO is stirred in Ru(bpy)_3_^2+^ solution, the redox peak current of Ru(bpy)_3_^2+^ increases with the increase of stirring time. After stirring for 8 min, the redox peak current reaches the maximum value. In addition, the peak current is significantly higher than that of n-SNA/ITO electrode. This shows that the positive probe can break through the electrostatic repulsion of the outer p-SNA in the stirring process. The probed can be enriched by the inner n-SNA, generating a high redox signal. Supplementary Figure S1 (SI) compares cyclic voltammetry curves obtained from bare ITO, n-SNA or bp-SNA modified ITO electrodes in KHP containing Fe(CN)_6_^3−^. As seen, the peak current obtained on n-SNA/ITO electrode decreased resulting from the permselective towards anionic Fe(CN)_6_^3−^. After the growth of bp-SNA, Fe(CN)_6_^3−^ is attracted by the electrostatic attraction of the external p-SNA layer and repelled by the electrostatic repulsion of the internal n-SNA layer. Thus, an increased current for Fe(CN)_6_^3−^ was observed at the bp-SNA/ITO compared with the n-SNA/ITO. However, the electrochemical signal of Fe(CN)_6_^3−^ displays almost no change after stirring for 30 min, indicating no effect on the enrichment of negative probes.

The electrochemical probe, methylene blue (MB), is chosen to be confined in the nanocage array in this work. In case of the positively charged MB, its electrochemical signals on ITO, n-SNA/ITO, bp-SNA/ITO electrodes exhibit a consistent trend with that of Ru(bpy)_3_^2+^. In the stirring state, MB can also break through the electrostatic repulsion of the outer p-SNA layer and be enriched by the negatively charged n-SNA. The enrichment equilibrium time was 6 min (Figure 3B).

**FIGURE 4:**
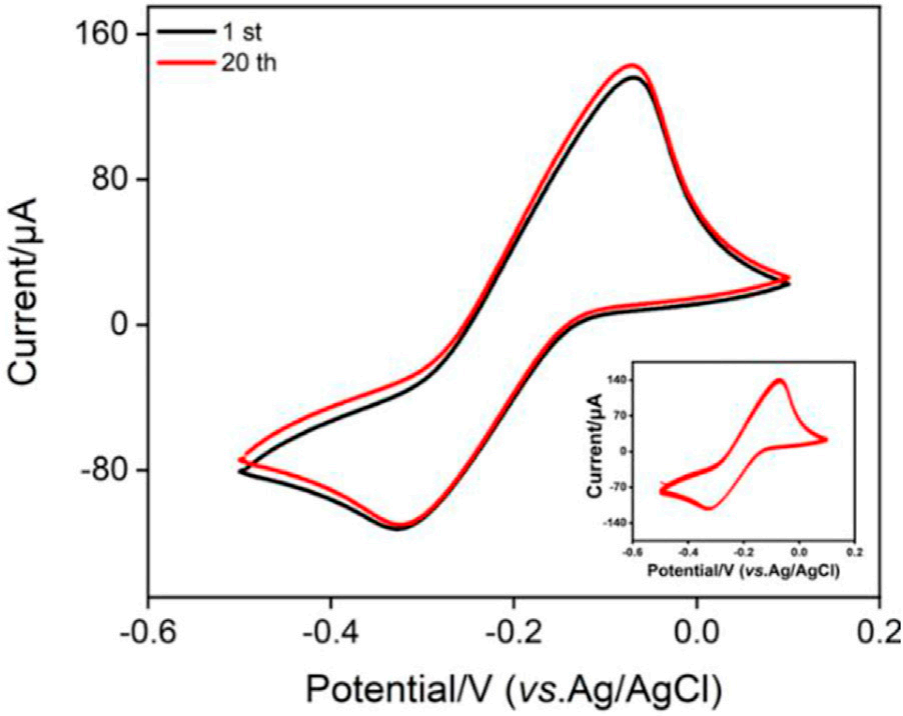
CV curves obtained on MB@bp-SNA/ITO in PBS (0.01 M, pH = 7.4). The inset is the CV curves obtained in successive scanning.

The inset in Figure 4 shows the CV curves when the electrode with fixed MB probe (MB/bp-SNA/ITO) is placed in buffer solution for continuous scanning. As seen, the peak currents do not significantly change when the electrode is continuously scanned. The peak currents obtained on the 1st and 20th scanning cycles are basically the same (Figure 4), indicating high stability of MB. This phenomenon is unlike the single-layered n-SNA modified electrode which only concentrates MB by electrostatic attraction. When MB is entrapped in electrostatic nanocage array in bp-SNA, the electrostatic attraction of the inner n-SNA and the electrostatic repulsion of the outer layer p-SNA makes MB stably confined in bp-SNA/ITO electrode.

### 3.4 Fabrication of the immunosensor

**FIGURE 5:**
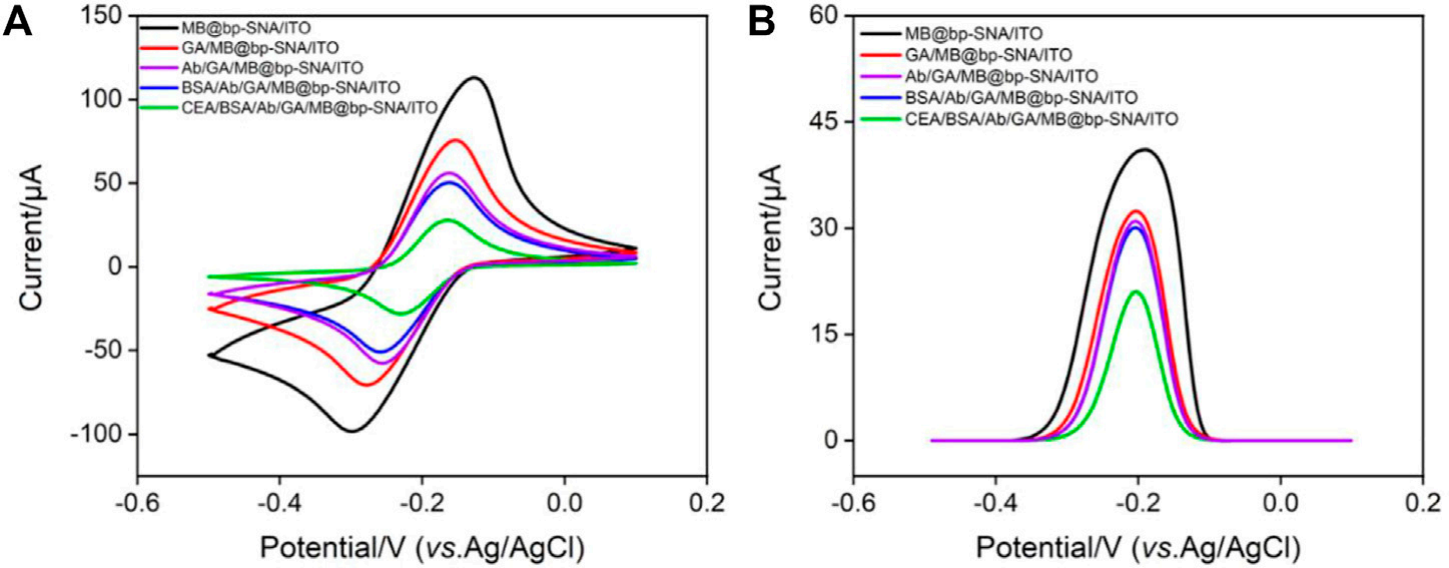
**(A)** CV curves and **(B)** DPV curves obtained on different modified electrode in PBS (0.01 M, pH = 7.4).

CV, DPV and EIS are used to observe the changes of electrode surface during the construction of immunosensor. When MB/bp-SNA/ITO is modified with GA derivatization (GA/MB/bp-SNA/ITO), immobilization of antibodies and blocking of non-specific sites (BSA/Ab/GA/MB/bp-SNA/ITO), the electrode surface resistance gradually increases, leading to gradually decreased redox peak current of MB (Figure 5A). The DPV curves in Figure 5B also proves the same result. When the fabricated immunosensor was incubated with CEA, decreased peak currents are observed resulting from the formation of antigen-antibody complex. Supplementary Figure S2 shows the EIS responses of each electrode to 2.5 mM Fe(CN)_6_^3−/4−^. As seen, the charge transfer resistance was increased after each modification, which was consistent with the current variation of CV and DPV responses.

### 3.5 Reagentless electrochemical detection of CEA using the constructed immunosensor

**FIGURE 6:**
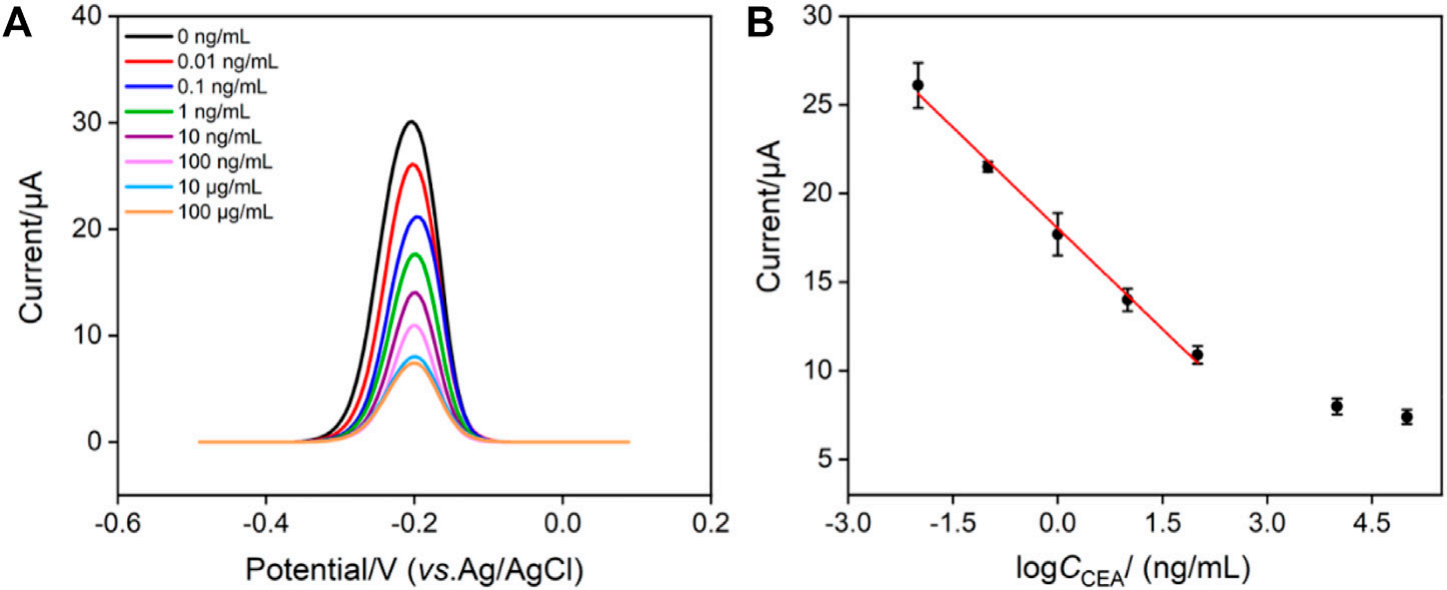
**(A)** DPV response of the immunosensor to different concentrations of CEA from 0.01 ng/mL to 100 μg/mL. **(B)** Calibration curve of the proposed EC immunosensor. Error bars represent the relative standard deviation (RSD) of three measurements.

The fabricated immunosensor, BSA/Ab/GA/MB/bp-SNA/ITO, is employed to detect CEA. Figure 6A shows the DPV curves obtained in presence of different concentrations of CEA. As shown, the peak current gradually decreases with the increase of CEA concentration because the formation of antigen-antibody complex hindered the electrochemical process of confined MB. The peak current (*I,* μA) is linear proportional to the logarithm of concentration of CEA (log*C*_CEA,_ ng/mL) in the range from 10 pg/mL to 100 ng/mL (*I* = −3.79 log*C*_CEA_ + 18.0, *R* ^2^ = 0.995, Figure 6B). The limit of detection (LOD) of 4 pg/mL is obtained at a signal-to-noise ratio of 3. The developed immunosensor has advantages of simple operation because of no need of antibody labeling or fabrication of sandwich immunocomplex. However, it cannot be applied under strong alkaline solutions owing to the possible hydrolysis of silica nanochannels.

**FIGURE 7:**
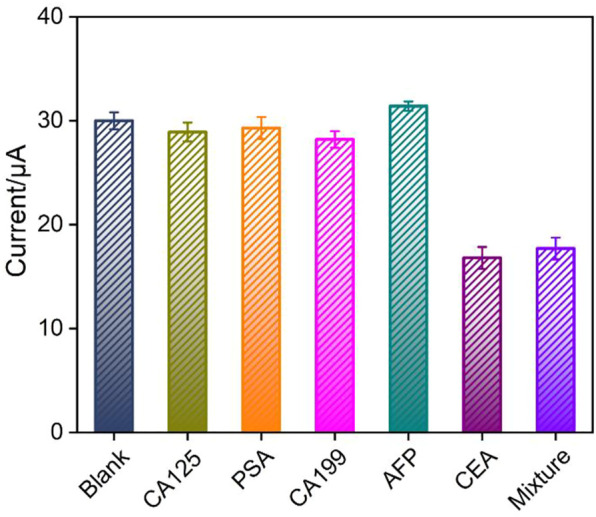
The peak current (*I*) obtained on the immunosensor in absence or presence of different interfering substances or their mixture. Error bars represent the RSD of three measurements.

The selectivity of immunosensor for the detection of CEA was investigated using tumor biomarkers including CA125, PSA, CA199, or AFP as possible interfering substances. Figure 7 shows the DPV peak current (*I*) obtained on the immunosensor in absence or presence of different interfering substances or their mixture. As seen, the peak current only decreases in the presence of CEA or mixture containing CEA, proving that the sensor has good detection selectivity. The immunosensor remains 92% of initial signal after 20-day storage period at 4°C, indicating high stability of the immunosensor.

### 3.6 Real sample analysis

The reliability of the fabricated immunosensor is evaluated by detection of CEA in real serum sample. The concentration of CEA in a healthy human serum (woman) detected by the constructed immunosensor is 1.92 ± 0.03 ng/mL (mean ± SD, *n* = 3), which is quite closed to that obtained using the commonly electrochemiluminescence (ECL) analyzer (1.95 ± 0.04 ng/mL). The obtained F-value (2.45) was lower than the critical F-value (19.00), suggesting no significant difference between these two data. To investigated the anti-interference or anti-fouling performance in complex biological media, the concentration of CEA in human serum is also detected by standard addition method. As shown in Supplementary Table S1 (SI), the recovery rate is between 97.7%–102% with low relative standard deviation (RSD, less than 4.0%). These results confirm high anti-interference or anti-fouling performance in complex biological media, suggesting good accuracy in CEA determination in real samples.

## 4 Conclusion

In summary, a probe-integrated electrochemical immunosensor is conveniently constructed for reagentless and sensitive detection of a tumor marker, carcinoembryonic antigen (CEA). To avoid adding any solution-based electrochemical probes, the redox probe, methylene blue (MB), is confined to the electrostatic nanocage array on the electrode surface. Electrostatic nanocage array is prepared by successive growing a layer of negatively charged nanochannel array film (n-SNA) and then a layer of positively charged amino-modified SNA (p-SNA). These two SNA layers with opposite charges form a bipolar film (bp-SNA) with electrostatic nanocage array to immobilize MB. When MB is confined, it is attracted by n-SNA and repelled by p-SNA, leading to high stability. The immunosensor can be constructed after the antibody is covalently immobilized followed with blocking the non-specific sites using bovine serum albumin (BSA). Electrochemical determination of CEA is realized by the reduced electrochemical signal of MB resulting from the formation of antigen-antibody complex and the increased surface resistance of the electrode. Although SNA has been widely used in electrochemiluminescence (ECL) systems because of high sensitivity. SNA-based electrochemical biosensors have advantages of simple instrument, low probe cost, easy integration and miniaturization for portable and real-time detection. The probe-integrated immunosensor has advantages of simple fabrication, no need of labelled antibody and formation of sandwich immunocomplexes, showing great potential in bioanalysis of tumor markers.

## Data Availability

The original contributions presented in the study are included in the article/Supplementary Material, further inquiries can be directed to the corresponding author.
