# Supplementary material for: Probe-integrated electrochemical immunosensor based on electrostatic nanocage array for reagentless and sensitive detection of tumor biomarker
Source: Front Chem. 2023 Mar 10;11:1121450. doi: 10.3389/fchem.2023.1121450 (PMC10036603; doi:10.3389/fchem.2023.1121450)
Supplement: Supplementary file 1 [file DataSheet1.PDF]

Table 1. Determination of CEA in human serum samples.

Sample	Spiked (ng/mL)	Found (ng/mL)	RSD (%)	Recovery (%)
Human serum[a]	0.0100	0.0100	1.9	100
	0.100	0.0977	2.3	97.7
	1.00	1.02	4.0	102

[a] The original concentration of CEA is 1.92 ng/mL. Serum samples were diluted 50 times after artificial addition of CEA. The indicated concentration of CEA is obtained after dilution with deduction of inherent CEA.

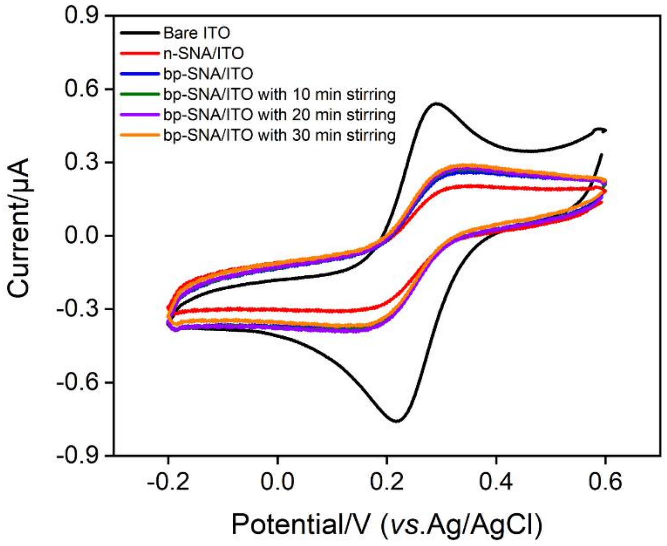

Figure S1. Cyclic voltammetry (CV) curves of bare ITO, n-SNA/ITO, bp-SNA/ITO electrodes in KHP (0.05 M, pH=4) containing 10 μM $K_3[Fe(CN)_6]$.

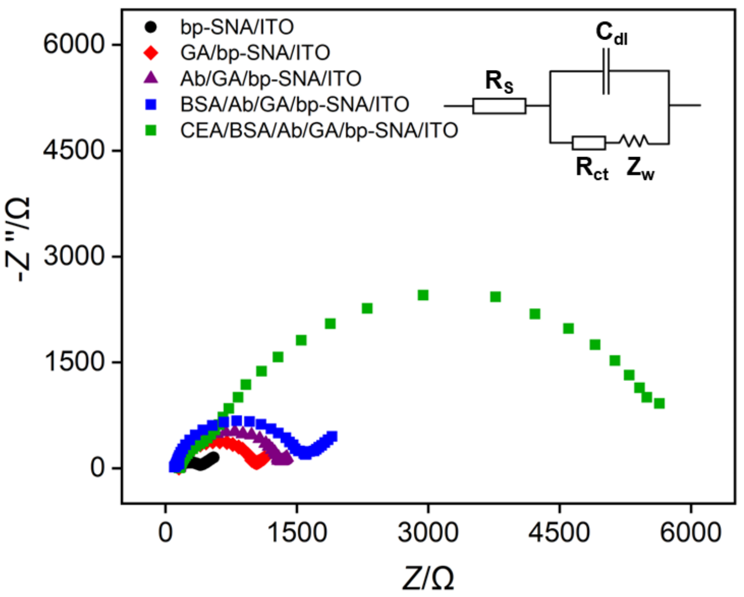

Figure S2. Electrochemical impedance spectroscopy (EIS) curves obtained on different electrodes. The electrolyte solution is 2.5 mM $Fe(CN)_6^{3-/4-}$ containing 0.1 M KCl.
